# A Zika Vaccine Targeting NS1 Protein Protects Immunocompetent Adult Mice in a Lethal Challenge Model

**DOI:** 10.1038/s41598-017-15039-8

**Published:** 2017-11-07

**Authors:** Aaron C. Brault, Arban Domi, Erin M. McDonald, Dalit Talmi-Frank, Nathanael McCurley, Rahul Basu, Harriet L. Robinson, Michael Hellerstein, Nisha K. Duggal, Richard A. Bowen, Farshad Guirakhoo

**Affiliations:** 1 Division of Vector-Borne Diseases, Centers for Disease Control and Prevention, Fort Collins, CO United States; 2 Geovax, Smyrna, GA United States; 3 Department of Biomedical Sciences, Colorado State University, Fort Collins, CO United States

## Abstract

Zika virus (ZIKV) is a mosquito-borne flavivirus that has rapidly extended its geographic range around the world. Its association with abnormal fetal brain development, sexual transmission, and lack of a preventive vaccine have constituted a global health concern. Designing a safe and effective vaccine requires significant caution due to overlapping geographical distribution of ZIKV with dengue virus (DENV) and other flaviviruses, possibly resulting in more severe disease manifestations in flavivirus immune vaccinees such as Antibody-Dependent Enhancement (ADE, a phenomenon involved in pathogenesis of DENV, and a risk associated with ZIKV vaccines using the envelope proteins as immunogens). Here, we describe the development of an alternative vaccine strategy encompassing the expression of ZIKV non-structural-1 (NS1) protein from a clinically proven safe, Modified Vaccinia Ankara (MVA) vector, thus averting the potential risk of ADE associated with structural protein-based ZIKV vaccines. A single intramuscular immunization of immunocompetent mice with the MVA-ZIKV-NS1 vaccine candidate provided robust humoral and cellular responses, and afforded 100% protection against a lethal intracerebral dose of ZIKV (strain MR766). This is the first report of (i) a ZIKV vaccine based on the NS1 protein and (ii) single dose protection against ZIKV using an immunocompetent lethal mouse challenge model.

## Introduction

First isolated in 1947 in Uganda, Zika virus (ZIKV; genus *Flavivirus*, family *Flaviviridae*) was associated with only occasional cases of febrile disease and considered of minor public health significance. In 2007, the first large epidemic of ZIKV was recorded on Yap Island in Micronesia, followed by a 2013 outbreak in French Polynesia. These events raised the profile of ZIKV as an emerging infectious disease^1^. Since its appearance in Brazil in late 2014, the virus has rapidly spread through the Americas in areas with competent populations of *Aedes* mosquito species. Subsequently, ZIKV has been implicated in human-to-human sexual transmission^2^, neurological manifestations including microcephaly in infants^3^, and Guillain-Barré syndrome (GBS) in adults^1^. In February of 2016, the World Health Organization (WHO) declared the ZIKV outbreak a “Public Health Emergency of International Concern”; as of this writing, the global risk assessment for ZIKV infections has not changed due to continued expansion of ZIKV to new geographical areas, where competent vectors are present.

ZIKV is transmitted to humans principally by infected *Aedes aegypti* and *Aedes albopictus* mosquitoes, the same species that transmit dengue (DENV1-4) and chikungunya viruses. Phylogenetic analyses of ZIKV demonstrate 2 major lineages: African and Asian, with >96% amino acid sequence identity^4^, constituting a single serotype^5^. The Asian lineage has been responsible for all ZIKV outbreaks in the Pacific and the Americas. Given the rapid geographic spread and likely potential for continued autochthonous transmission of ZIKV throughout the Americas, a vaccine is urgently needed to provide protection from ZIKV disease and ZIKV congenital syndrome (ZCS).

Antibody-Dependent Enhancement (ADE) of viral infection has been documented *in vitro* and *in vivo* as a potential risk with ZIKV structural proteins (prM/E)-based vaccines^6–8^. This is especially relevant for cross-reactive antibodies between DENV and ZIKV, because DENV seroprevalence is >90% in many parts of the Americas affected by ZIKV^9^. Since antibodies binding to prM or E proteins of ZIKV or DENV have been shown to increase infection of monocytes through Fc gamma receptors^7^, there is a risk that DENV antibodies could contribute to more severe ZIKV infections and/or ZCS or ZIKV antibodies from immunization could enhance DENV disease. Until larger-scale Phase II-III clinical studies with ZIKV prME immunogens have been performed to evaluate the threat of ADE in dengue endemic areas (e.g. enhancement of DENV infections by ZIKV immunity or the potential for adverse effecs of live attenuated ZIKV vaccine due to pre-existing dengue immunity), ADE will remain a concern for use of these vaccines in the populations most in need of ZIKV immunization. While the E protein is commonly considered as a desired antigenic target for eliciting protective neutralizing antibodies against ZIKV, nonstructural protein-1 (NS1) has been shown to induce protective non-neutralizing antibodies that target virus-infected cells through Antibody-Dependent Cellular Cytotoxicity (ADCC) and complement-dependent pathways^10,11^. Therefore, NS1 protein and anti-NS1 antibodies have been proposed as flaviviral vaccines and therapeutic candidates, respectively^10,12,13^ (see also the supplementary text). Unlike potential enhancement of infection between DENV and ZIKV anti-prME antibodies^7,14^, anti-NS1 antibodies should not pose a risk of ADE to vaccinated individuals since NS1 proteins are not packaged with the virus or found on the surface of virions^15^.

Here we describe the generation of a ZIKV vaccine based on delivery of the NS1 protein by a recombinant Modified Vaccinia Ankara (MVA) vector which has previously induced robust and durable protective immunity in pre-clinical and clinical HIV vaccine trials^16,17^ and preclinical Ebola studies^18^. Immunocompetent mice were immunized with the NS1 vaccine, and immunogenicity and protective efficacy were assessed in a newly developed lethal intracranial challenge model^19^.

## Results

### Construction and characterization of MVA-ZIKV-NS1 vaccine

**Figure 1:**
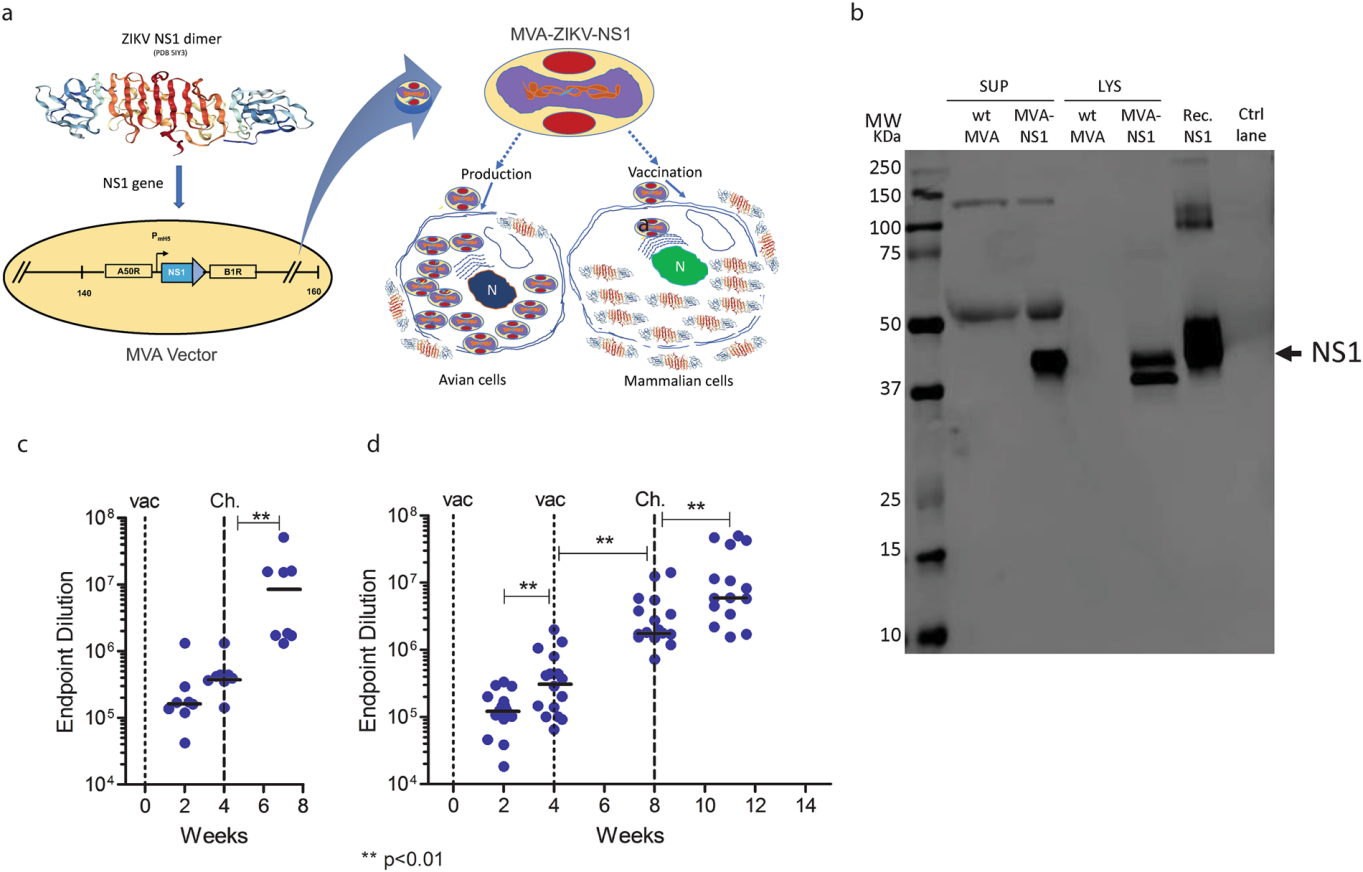
Construction, Western blot, and immunogenicity of MVA-ZIKV-NS1 vaccine in CD-1/ICR mice following single or prime-boost immunization. (**a**) ZIKV NS1 gene (Suriname 2015 isolate Z1106033) was inserted into the MVA restructured and modified deletion III. This insertion site has been identified to support high expression and insert stability. P_mH5_, modified H5 promoter. Numbers are coordinates in the MVA genome. MVA-ZIKV-NS1 is replication competent in avian cells (producing both MVA and inserted transgenes) but is replication deficient in mammalian cells producing mainly inserted transgenes (e.g. NS1) but not infectious MVA viruses. Image of the ZIKV NS1 dimer is from RCSB PDB (www.Rcsb.org) of PDB ID 5IY3^41^ (**b**) Western blot, DF1 chicken fibroblasts cells were infected with either wild type MVA (wt MVA) or MVA-ZIKV-NS1 (MVA-NS1) at multiplicity of infection (MOI) of 0.1. The expression of full-length NS1 was confirmed using anti-ZIKV-NS1-protein mouse monoclonal antibody (IgG1). Recombinant ZIKV NS1 protein (Rec. NS1) served as a positive control. A loading control lane (Ctrl lane) served as another negative control. Like other flaviviral NS1 proteins, ZIKV NS1 obtained from infected cell lysates (LYS) migrates as a doublet (intracellular NS1 (non-glycosylated, lower band) and cell-surface NS1 (glycosylated, upper band)^20^. Only fully glycosylated NS1 was found in the supernatants (SUP). (**c**) Single dose group, endpoint dilution geometric mean titer (GMT) determined by ELISA using sera from mice obtained at 2 and 4 weeks post-immunization (vac) and 3 weeks post-i.c. challenge (Ch) with 10^5^ PFU of MR766. (**d**) Prime-boost group, endpoint dilution GMT determined by ELISA using sera from mice obtained at 2 and 4 weeks (post-prime) and at 4 weeks (post boost) immunization and 3 weeks post i.c. challenge with 10^5^ PFU of MR766.

We generated a ZIKV vaccine (MVA-ZIKV-NS1) (Fig. 1a) by inserting sequences of the NS1 gene of a 2015 Asian isolate (Suriname) into the MVA vector. Like other flaviviral NS1 proteins, ZIKV NS1 obtained from infected cell lysates migrates as a doublet (intracellular NS1 non-glycosylated, lower band) and cell-surface NS1 (glycosylated, upper band)^20^. Only fully glycosylated NS1 was found in the supernatants (MW of ~45 KDa) (Fig. 1b).

### Protective immunity

Immunogenicity and protective efficacy were assessed in a newly developed lethal intracerebral (i.c.) challenge model^19^. Since current immunocompetent mouse models for ZIKV are not susceptible to lethal outcome, and type I/II interferon receptor-knockout mice used extensively as a challenge model for ZIKV vaccine candidates do not recapitulate normal immune responses of human vaccinees, our newly developed i.c. challenge model using a neurovirulent African strain of ZIKV (MR766) represents a stringent test of immunity in a normal mouse. Outbred CD-1/ICR mice were immunized by the intramuscular (i.m.) route with either MVA-ZIKV-NS1 or phosphate buffered saline (PBS) following a prime-only or a prime-boost regimen, challenged i.c. with MR766, and observed for weight loss and overt signs of illness.

**Figure 2:**
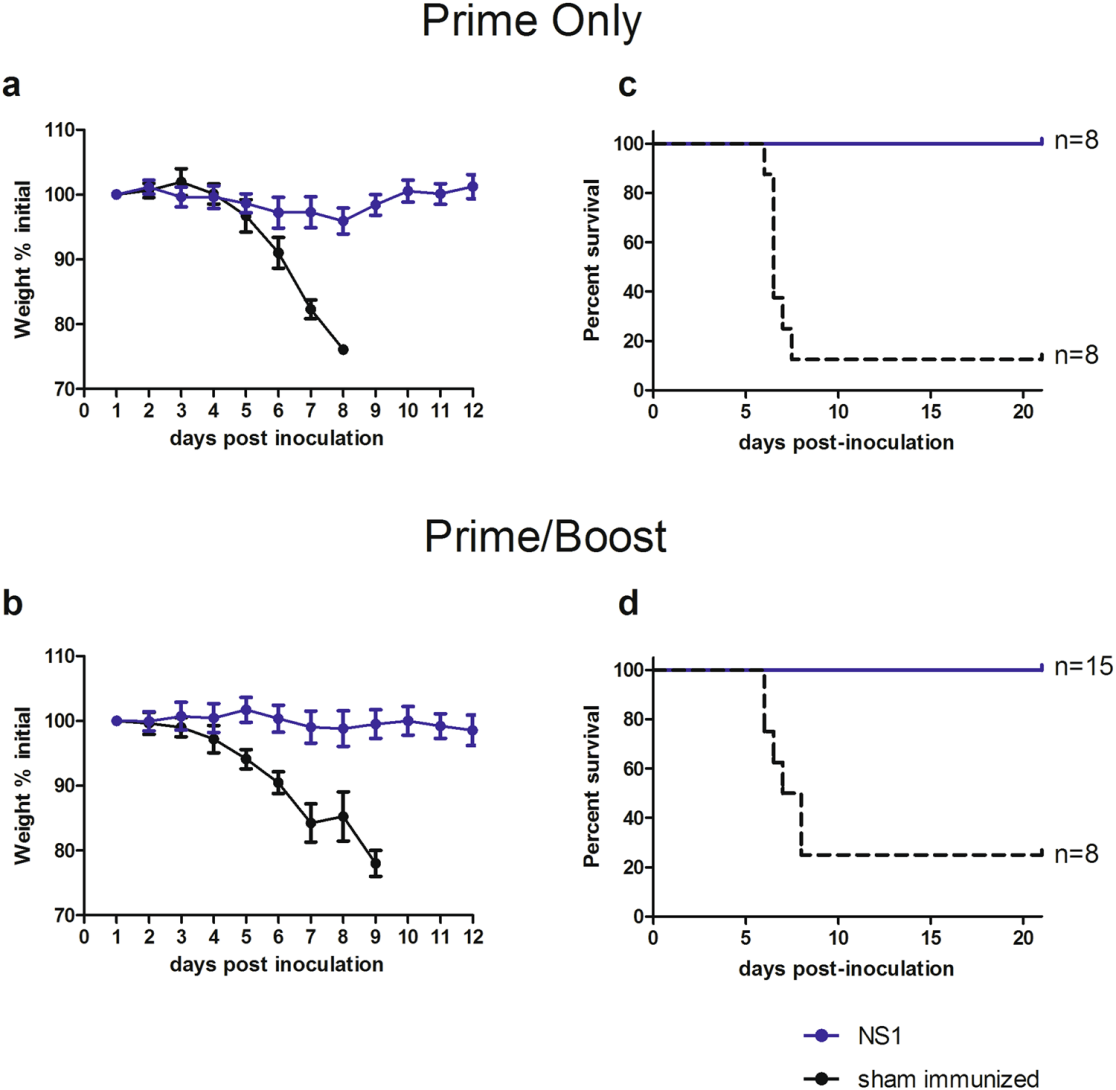
Survival of MR766 challenged MVA-ZIKV-NS1 immunized and sham-immunized mice (**a**–**d**). Mice were challenged i.c. with 10^5^ pfu ZIKV (MR766) 28 days after immunization. **(a)** Mice immunized by single immunization only or **(b)** Prime-Boost regimen, maintained weight (blue) compared to sham-immunized controls (black). **(c)** Complete protection was afforded by either single immunization or (**d)** through the prime-boost regimen while controls demonstrated 87.5% and 75% mortality, respectively **(c**,**d)**.

**Figure 3:**
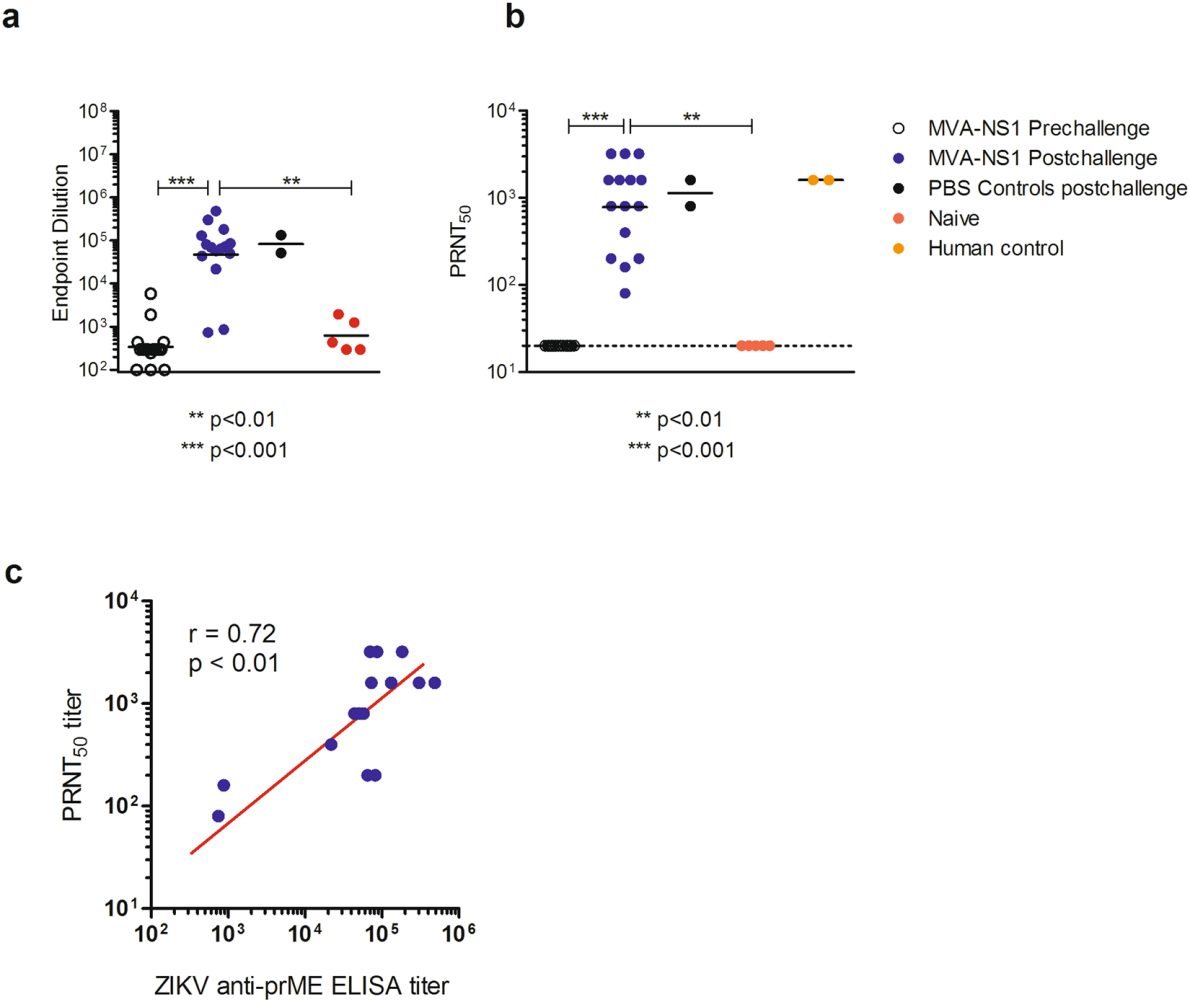
Anti-prME responses pre- and post-challenge. (**a)** Binding antibodies were determined by ELISA using prME Cos-1 antigen. **(b)** Neutralizing antibody titers were determined by plaque reduction neutralization assay in MVA-ZIKV-NS1 vaccinated mice pre-and post-challenge. **(c)** Correlation of post-challenge anti-prME ELISA titer and PRNT_50_ titers in MVA-ZIKV-NS1 vaccinated mice.

All vaccinated mice demonstrated robust NS1-specific antibody responses, as measured by ELISA, as early as 2 weeks post-immunization; responses increased 2 weeks later and following booster immunization (p < 0.01) (Fig. 1c,d). Upon challenge, immunized mice were fully (100%) protected after both prime-only and prime-boost immunizations (Fig. 2a–d). No significant symptoms or weight loss were observed in any vaccinated animal. In contrast, most sham-immunized animals lost weight (Fig. 2a and b), demonstrated signs of neurological disease, and were euthanized according to approved IACUC protocols (~70–80% mortality). The titer of anti-NS1 antibodies in single and prime-boost regimen increased after challenge 14.3- and 3.1-fold, respectively (Fig. 1c and d), indicating that the prime-boost vaccination series might have limited challenge virus replication more effectively than a single immunization. Moreover, the African challenge virus induced *de novo* a high level of anti-E antibodies that bound and neutralized the heterologous Puerto Rico strain isolated in 2015 (PRVABC59) comparable to the level that was observed with the 2 unvaccinated mice that survived the challenge and human ZIKV positive sera (Fig. 3a and b), confirming that ZIKV constitutes a single serotype^5^. There was also a correlation between the level of binding (ELISA) and neutralizing antibodies post-challenge in mice (Fig. 3c), consistent with recent observations of ZIKV infection in rhesus monkeys^21^.

### Functional activities of anti-NS1 antibodies and T cell responses

**Figure 4:**
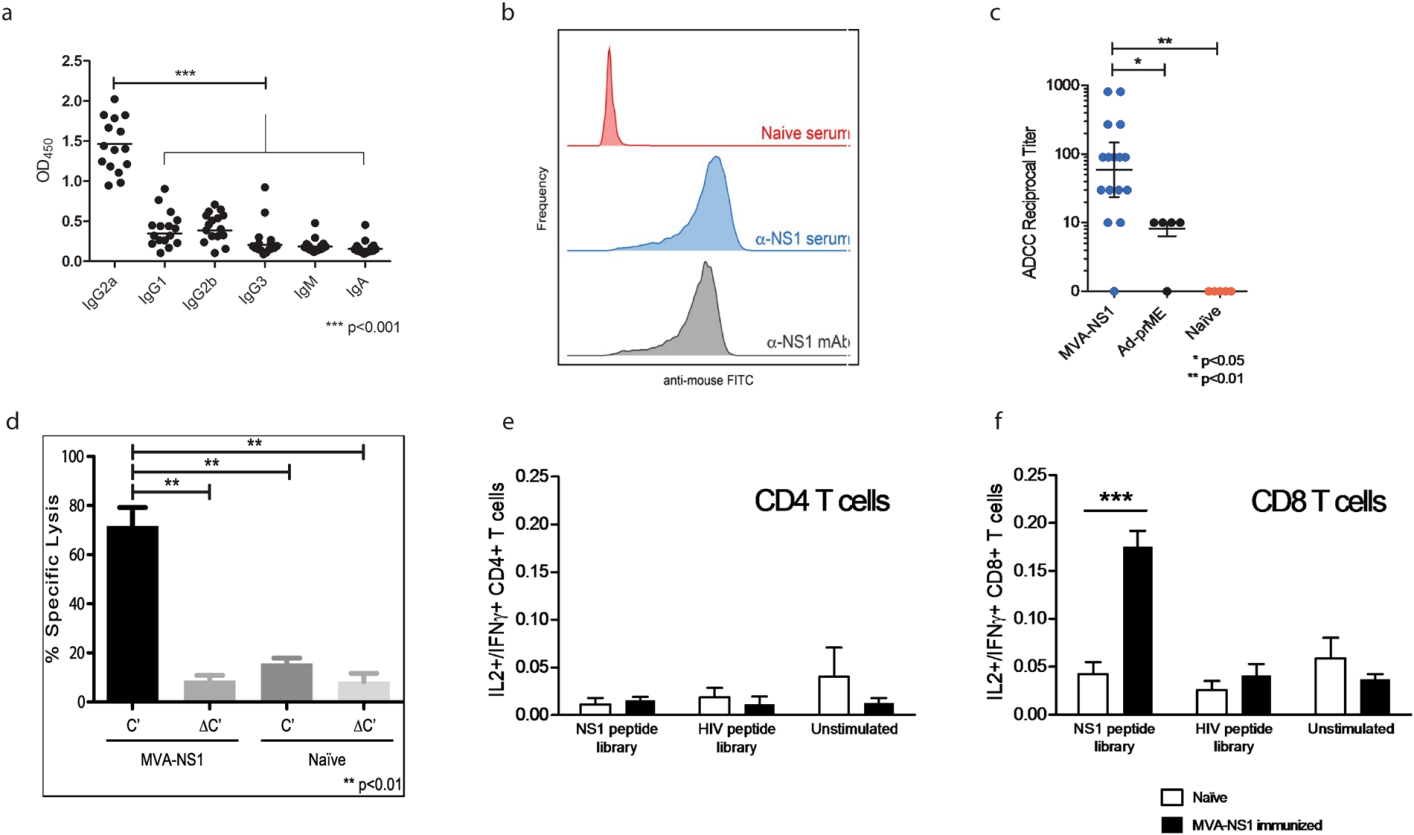
IgG Isotype specificity, surface expression of NS1 proteins, ADCC, complement fixation, and T cell responses of MVA-ZIKV-NS1 antisera. (**a**) The magnitude of Ig isotype specificity of immune sera from MVA-ZIKV-NS1 immunized mice was determined by ELISA, using rabbit anti-mouse immunoglobulin isotype-specific antibodies recognizing IgG1, IgG2a, IgG2b, IgG3, IgM, or IgA antibodies. (**b**) NS1-expression of inoculated Vero cells assayed by flow cytometric analysis. (**c**) ADCC activity observed following incubation of ZIKV-infected Vero cells (target cells) with serial dilutions of immune sera generated from MVA-ZIKV-NS1, Adeno-prME VLPs^24^, or naïve sera in the presence of effector cells (Jurkat cell line). (**d**) Complement-mediated lysis of ZIKV-infected Vero cells. C′, native complement, ΔC′, heat inactivated complement. Naïve sera, sera from sham immunized mice. (**e**,**f**) T cell responses of immunized mice. (**f**) ICS analysis of CD8+ T cells demonstrated IL2 and IFNγ production in the CD8+ T cell subset 10 days post-vaccination. (**e**) No appreciable cytokine production was observed in CD4+ T cells by this time.

Since NS1 proteins are not present on incoming virions (challenge virus), it is not expected that anti-NS1 antibodies provide sterile immunity. Therefore, NS1 is likely to protect through at least two different mechanisms: i) Fc-mediated non-neutralizing antibodies that bind virus-infected cells displaying NS1 proteins, leading to cell death through complement fixation, ADCC, and phagocytosis; and ii) antigen-specific CD8^+^ T cells targeting NS1 epitopes of infected cells in an MHC I restricted manner. Anti-mouse-IgG1, -IgG2a, -IgG2b, -IgG3, -IgM, and -IgA isotyping demonstrated that the anti-NS1 humoral response was predominantly comprised of IgG2a (p < 0.001) (Fig. 4a), a hallmark of a dominant Th1 lymphocyte populations, important for mediating complement and ADCC activities observed with protective flavivirus anti-NS1 polyclonal and monoclonal antibodies in mice^11,22,23^. Protection by anti-NS1 IgG2a has been shown to target NS1 proteins displayed on infected cells and to recruit lymphoid cells, bearing Fc-γ receptors, to kill infected cells through ADCC^11^. Given that NS1 is not present on the surface of virions, yet is displayed on the surface of virus-infected cells, we hypothesized that a protective humoral response would be initiated by the Fc portion of anti-NS1 antibodies. To address this, we tested NS1 immune sera for ADCC activity using ZIKV infected Vero cells in an ADCC Reporter Bioassay where binding of the Fc portion of antibody to the FcγRIIIa of the effector cells (Jurkat) results in a quantifiable luminescence signal from NFAT (nuclear factor of activated T-cells) pathway (ADCC Reporter Bioassay, Complete Kit WIL2-S from Promega) (see also Method section). Flow cytometry using sera from MVA-ZIKV-NS1 immunized mice verified the expression of ZIKV NS1 in Vero cells at 48 hours post-inoculation (Fig. 4b). ADCC activity was demonstrated (Fig. 4c) in the sera of 15 out of 16 NS1-immunized mice, compared to 5 control sera (generated by an Adeno-ZIKV prME VLP vaccine)^24^ having GMT of 31,657 and 1,056 in ELISA and PRNT_50_, respectively; data not shown) (p < 0.05). An assay was also developed to test the ability of immune sera to utilize complement fixation for the killing of virus-infected cells. The MVA-ZIKV-NS1 sera, but not the naïve sera, clearly showed specific lysis in the presence of complement (Fig. 4d) (p < 0.01). This was consistent with elicited antibodies being predominantly of the IgG2a isotype (Fig. 4a).

At ten days post-immunization, NS1-specific CD8+ T cells were raised in immunized mice (Fig. 4f). Intracellular cytokine staining analysis of splenocytes taken from immunized mice 10 days after a single vaccination with MVA-ZIKV-NS1 demonstrated a significant number (p < 0.001) of IFNγ- and IL2-expressing CD8^+^ T cells in response to stimulation with a ZIKV NS1 peptide library, but not in response to negative control peptides (Fig. 4f). Consistent with previous reports^21^, NS1-specific CD4^+^ T cell responses were not observed above the limit of detection at this early time point (Fig. 4e).

### Clearance of ZIKV after challenge in brains of vaccinated mice

**Figure 5:**
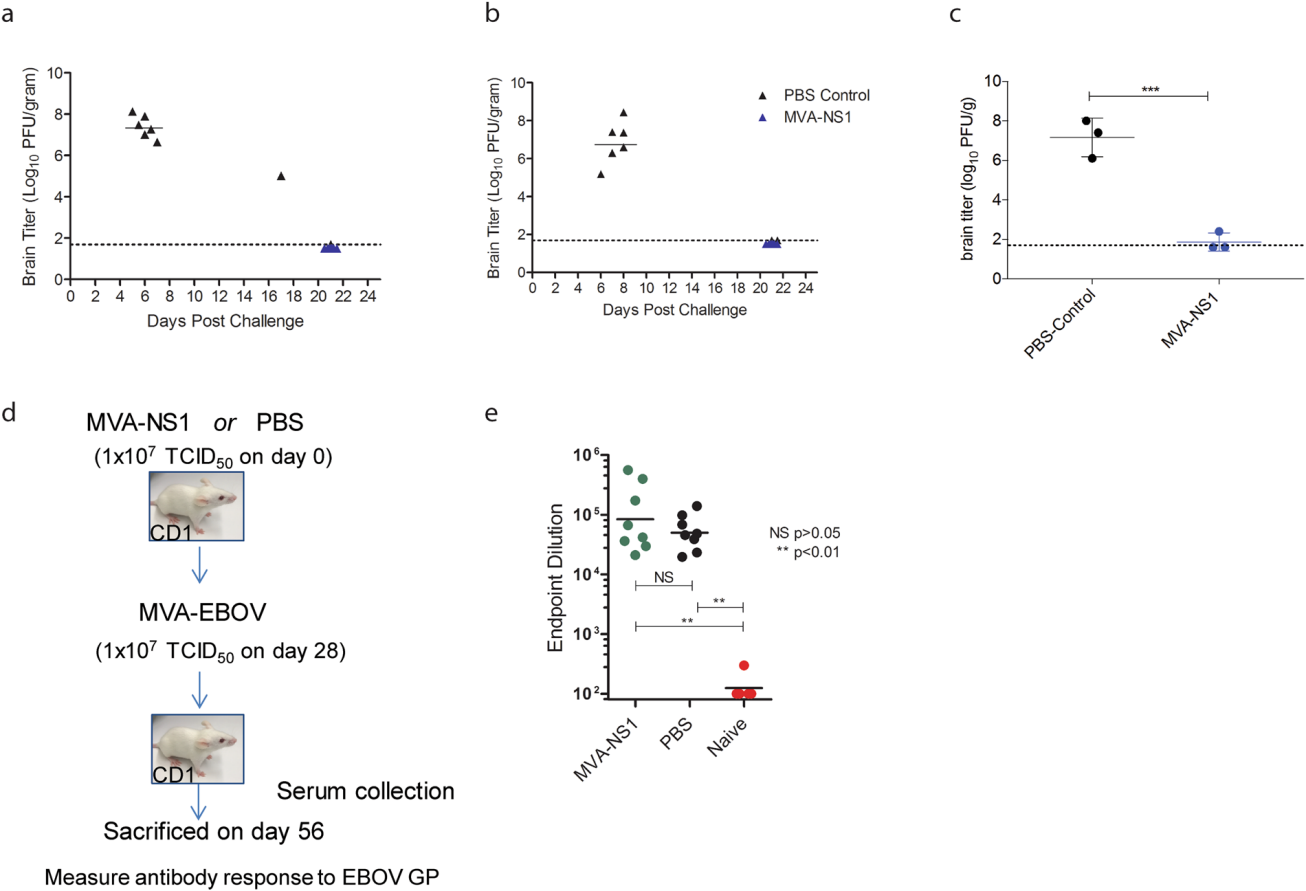
Determination of brain virus titers and vector immunity. (**a)** Brain titers after single and **(b)** prime-boost immunizations. Post-challenge, mice that displayed clinical signs of illness, including weight loss, were euthanized. All surviving mice were euthanized on Day 21 post-challenge. Brains were titrated on Vero cells by plaque assay to determine levels of ZIKV, as described previously^26^. Individual points are shown for each mouse. The limit of detection (LOD) was 1.7 log_10_ PFU/gram. Brain samples with undetectable viremia are shown at this limit. **(c)** Brain titers from singly immunized and control vaccinated mice at 5 dpi. **(d)** Mice were immunized with MVA-ZIKV-NS1 or with PBS, and subsequently (4 weeks later) immunized with MVA-EBOV^18^. **(e)** Four weeks post-MVA-EBOV immunization, mice were bled and assessed for EBOV GP endpoint titers by ELISA, and compared to naïve mouse sera.

To assure that ZIKV is cleared from brains of vaccinated animals, all MVA-ZIKV-NS1 immunized mice were sacrificed at the end of experiment (Day 21 post-challenge), and viral titers determined for brain homogenates using plaque assays^25^. In line with lack of weight loss, any adverse disease symptoms, and complete survival of vaccinated mice, we could not find any residual ZIKV at the end of observation period. In contrast, control mice died with a high central nervous system (CNS) viral (up to 8 log_10_ PFU/g tissue) between Days 5–8 post-challenge (Fig. 5a and b). In one of the control mice that survived until Day 17, a lower titer of virus (~5 log_10_ PFU/gram of tissue) was detected, whereas no virus was detected in the brain of the single surviving mouse in the control group euthanized on Day 21 (Fig. 5a and b). In a separate experiment, the brain titers at Day 5 post-challenge (previously determined as the peak virus titer)^26^ was determined in groups of 3 mice vaccinated with a single dose of the MVA-ZIKV-NS1 and compared to that of unvaccinated animals. The mean brain titer in i.c. challenged mice that had received a PBS immunization was 7.2 log_10_ PFU/g tissue. In contrast, no detectable viral load was observed in two of three MVA-ZIKV-NS1 immunized mice (<1.6 log_10_ PFU/g tissue). The mean viral load in the brains of MVA-ZIKV-NS1 immunized mice (1.9 log_10_ PFU/g tissue) was 200,000-fold lower than that observed in the PBS immunized mice (p > 0.001) (Fig. 5c) indicating a rapid clearance of the infectious virus in brains of vaccinated and challenged mice. We did not attempt to measure viremia in sera of mice post-challenge because as no peripheral viremia was previously found after i.c. inoculation with the MR766 virus^26^.

### Vector Immunity

To determine if pre-existing immunity to the MVA vector could have a detrimental effect on immunogenicity of subsequent MVA vaccinations, antibody titers against Ebola virus glycoprotein (EBOV GP) were assessed in mice sequentially vaccinated with MVA-ZIKV-NS1 and MVA-EBOV, previously shown to protect rodents and Rhesus macaques after a single dose vaccination^18^. Mice were vaccinated with 1 ×10^7^ TCID_50_ MVA-ZIKA-NS1 or PBS. At 8 weeks post-immunization, all mice were vaccinated with 1 × 10^7^ TCID_50_ MVA-EBOV. Four weeks later, animals were bled and antibody responses were measured against EBOV GP by ELISA (Fig. 5d). Despite previous MVA vaccination, no significant difference in endpoint dilution of anti-GP antibodies was observed (Fig. 5e).

## Discussion

We have utilized an MVA platform, previously tested in 4 clinical trials and shown to induce a durable anti-HIV immune response^16,17^, to produce an NS1-based vaccine against ZIKV. The sequences used in this vaccine construct were derived from the Asian strain isolate Z1106033 (Suriname) of the 2015 ZIKV epidemic, which is heterologous to the sequence of MR766 (an African strain isolated in 1947 in Uganda and passaged 149 times in suckling mouse brain) used for challenge^19^. Nevertheless, because ZIKV is considered as a single serotype (total sequence divergence not exceeding divergence within flavivirus species)^5^, all ZIKV viruses are equally neutralized by human convalescent sera^27^, and the sequence of the NS1 protein has 99.3% similarity among all ZIKVs^28^, this NS1-targeting vaccine should be effective against all ZIKV strains^19,27^.

Although NS1 is expressed on infected cells, it is not packaged into ZIKV virions^29^. NS1 was chosen based on documented evidence that flavivirus NS1 has been sufficient to elicit a protective immune response to other flaviviruses in murine models^10^. This novel combination of vector platform and native antigen conformation has the potential to yield a vaccine that would provide protection with no risk of ADE. The ideal ZIKV vaccine is safe for women of child-bearing age, immunocompromised individuals (e.g. HIV or patients on immunosuppressive drugs), children and the elderly, cost effective to manufacture, effective against all circulating ZIKV strains, and induces rapid onset of protective levels of antibody as well as T cell responses after a single dose. The MVA-ZIKV-NS1 vaccine is attractive for accelerated development because it provides the potential for a safe single dose elicitation of protective immune responses. MVA vaccines are replication competent in avian cells used for vaccine production, yet replication deficient in mammalian cells, making them putatively safe for humans, including immunocompromised or pregnant individuals. MVA has been shown to be safe in >120,000 vaccinees, including HIV-positive individuals, and has shown no reproductive toxicity in studies in pregnant rats^30^. For ZIKV vaccines, WHO has recommended non-live/inactivated approaches for vaccination of women of child-bearing age. The approach described herein is in accordance with this WHO recommendation, as MVA-ZIKV vaccines match the excellent safety profile of non-live/inactivated vaccines without the need for an adjuvant, and additionally offer the potential for high levels of immunogenicity and efficacy after a single dose. Moreover, an NS1 vaccine poses no predicted risk of induction of ADE in vaccinees living in areas endemic for DENV or other flaviviruses, which are the clear target market for a ZIKV vaccine. The potential risks of flaviviral immunization in DENV-endemic areas has been highlighted by the increased incidence of hospitalizations of seronegative children (younger than 9 years old) vaccinated with Dengvaxia®, indicating that the vaccine could induce ADE in flavivirus naïve children^31^. Though no direct data have yet been published indicating ADE effects in human vaccinees with a prME-targeted immune response, risk of this potential complication has been highlighted by studies in which passive transfer of DENV or WNV immune sera to immunocompromised mice resulted in more severe disease progression upon ZIKV infection^7^. Given this risk, alternative immunogenic targets that are not associated with ADE to heterologous flaviviral infections are attractive candidates for use in areas of high flaviviral endemicity.

The i.c. challenge model used for efficacy assessments in the present studies demonstrates that peripheral immunity against NS1 can protect against virus in the CNS, thus preventing neurological disease by clearing virus in the brain of challenged mice. Furthermore, this model has demonstrated that the immune responses generated against the NS1 protein sequences of a contemporary Asian genotype affords complete protection against a heterologous African ZIKV, in line with the evidence that ZIKV constitutes a single serotype^27^. Conversely, African strains induced cross-lineage efficacy against Asian strains in mice and monkeys^19,32^. The use of MR766, a neuroadapted African lineage, for challenge, is relevant and represents a stringent test of vaccine immunity, because both African and Asian lineage ZIKV strains have displayed similar inhibitory effects on neuronal differentiation and organoid development using human induced pluripotent stem cells^33^, and have both demonstrated infection of neural progenitor cells that could be differentially associated with induction of microcephaly^34^. Moreover, in *Stat2* ^−/−^ mice, which are highly susceptible to ZIKV infection and recapitulate virus spread to the CNS and other organs, African strains of ZIKV induced a higher level of inflammatory cytokines and severe neurological symptoms followed by death, compared to the Asian strains that rarely caused any lethality^35^. Since current immunocompetent mouse models for ZIKV are not susceptible to viremia or lethal outcome, and type I/II interferon receptor-knockout mice (e.g. AG129 mice) used extensively as a challenge model for ZIKV vaccine candidates, do not recapitulate normal immune responses of human vaccinees, our newly developed i.c. challenge model^19^ using a neurovirulent African strain of ZIKV, represents a stringent test of immunity in a normal mouse vaccinated by the i.m. route.

Under normal circumstances (e.g. humans bitten by ZIKV-infected mosquito), it is expected that a high level of protection against ZIKV infection will be afforded by MVA-ZIKA-NS1 immunization through humoral and cellular responses. The titer of anti-NS1 antibodies in single and prime-boost regimen increased after challenge 14.3- and 3.1-fold, respectively, indicating that the prime-boost vaccination series might have limited challenge virus replication more effectively than a single immunization. Moreover, the African challenge virus induced *de novo* a high level of anti-E antibodies that bound and neutralized the heterologous Puerto Rican strain comparable to the level that was observed with the 2 unvaccinated mice that survived the challenge and human ZIKV positive sera confirming that ZIKV constitutes a single serotype^5^. There was also a correlation between the level of binding (ELISA) and neutralizing antibodies post-challenge in mice, consistent with what has recently been observed with ZIKV infection of rhesus monkeys^21^. In line with lack of weight loss, any adverse disease symptoms and complete survival of vaccinated mice, we could not find any residual ZIKV at the end of observation day, whereas control mice died with a high burden of brain virus (up to 8 log_10_ PFU/g of tissue) between Days 5–8 post-challenge. It appears that the virus clearance in brains of vaccinated mice occurred at ≤5 days post-challenge.

The strong NS1-specific CD8^+^ T cell response from single dose vaccinated mice, most likely contributed to clearance of the virus from the brain after the i.c. challenge, as has been shown with yellow fever and West Nile virus infected mice^21,36^. The lack of any significant CD4^+^ T cell response in our experiment on Day 10 was consistent with a report where appearance of CD4^+^ T cell response in ZIKV infected Rhesus monkeys was not observed until after production of antibody and CD8^+^ responses, at 2 weeks post-infection^21^.

We did not attempt to measure viremia in sera of mice post-challenge because i) we did not want to put animals under additional stress by bleeding procedures which could adversely affect their survival outcome, and ii) no peripheral viremia could previously be found after the i.c. inoculation with the MR766 virus^19^. Since the protection of an NS1-based vaccine requires some levels of virus replication post-challenge (to express NS1 antigens targeted for antibody and T cell immune responses), future studies will include tests for prevention of viremia and protection of immunized immunocompetent mice administered type-I anti-interferon receptor monoclonal antibody prior to subcutaneous challenge with ZIKV.

NS1 has been shown to play a critical role in enhancing *Ae*. *aegypti* oral susceptibility^37^, and a mutation in NS1 gene (A188V) of the emergent Asian genotype associated with higher levels of secreted NS1 has been proposed to have contributed to further enhancement of oral susceptibility^38^. Furthermore, polyclonal sera generated against NS1 and administered in infectious blood meals was demonstrated to attenuate ZIKV mosquito infectivity^38^. Therefore, we expect that our NS1-based vaccine could not only protect individuals against symptomatic ZIKV disease, without any risk of ADE, but could also reduce infection rates in the mosquito vector, thus interrupting ZIKV transmission in areas of high endemicity with lower vaccine coverage.

## Materials and Methods

### Cell Lines

Vero cells were obtained from ATCC and were authenticated by COI assay. Mycoplasma contamination was determined by Hoechst DNA stain, Agar culture, and PCR. Chicken embryo fibroblasts (CEF) were obtained from Charles RiverLaboratories. Mycoplasma contamination was determined by serum plate agglutination.

### Construction of vaccine viruses

The MVA-vectored ZIKV vaccine candidate (MVA-ZIKV-NS1) expressing the ZIKV NS1 protein was constructed using shuttle vectors originally developed in the laboratory of Dr. Bernard Moss. These shuttle vectors have proven to yield stable vaccine inserts with high, but non-toxic, levels of expression with HIV and hemorrhagic fever virus vaccine candidates^39,40^. The NS1 sequence (strain Suriname 2015, Genbank KU312312) was inserted into a restructured and modified deletion III between the A50R and B1R genes (Fig. 1a). The NS1 gene sequence was codon optimized for MVA with synonymous mutations introduced to interrupt homo-polymer sequences (>4 G/C and >4 A/T) to reduce RNA polymerase errors that could lead to frameshifts. Inserted sequences were edited for vaccinia-specific terminators to remove motifs that could lead to premature termination^40^. All vaccine inserts were placed under the modified H5 early/late vaccinia promoter as described previously. The recombinant virus was rescued by homologous recombination into CEF cells infected with MVA parental virus and transfected with MVA shuttle virus expressing ZIKV NS1 under the control of a vaccinia virus early-late promoter. The shuttle plasmid also expresses a green fluorescent protein (GFP) as reporter. The ZIKV NS1 and GFP are flanked by MVA sequences needed for recombination. The GFP expressing virus was clone purified by limiting dilutions until cleaned from the parental virus. Cloning was continued until the GFP has been removed by homologous recombination. The virus was then scaled up in CEF cells. All cloning and virus production work were performed in a dedicated room at GeoVax, with full traceability and complete documentation of all steps using Bovine Spongiform encephalopathy/Transmissible Spongiform Encephalopathy (BSE/TSE)-free raw materials so the virus stock can be directly used for production of a Pre-Master virus seed during cGMP manufacturing.

### Western blots

DF1, continous chicken fibroblasts, cells were infected with MVA-ZIKV-NS1 at multiplicity of infection (MOI) of 0.1. Cell lysates and supernatants were harvested at 48 hrs post-infection and loaded onto 12.5% SDS gel; the expression of full-length NS1 was confirmed by Western blot using anti-ZIKV-NS1-protein mouse monoclonal antibody (IgG1) from Aalto Bio Reagents, Catalog # AZ1225. Recombinant ZIKV NS1 from Sino Biological served as a positive control (Fig. 1b).

### Preparation of Stock viruses for vaccination

MVA-ZIKV-NS1 vaccine stocks were produced in primary CEF obtained from Charles River Laboratories. Briefly, cells were inoculated at an MOI of 0.01, infected cells harvested at 3 days PI, and viruses were purified using sucrose gradient centrifugation. Titers were determined as TCID_50_ by limiting dilution in DF1 cells. Potency was measured by immunostaining plaques on DF1 cells inoculated with vaccine candidates. Plaques were stained with antibodies specific for ZIKV NS1 protein (Aalto Catalog # AZ1225) and MVA (provided by Dr. Moss).

### Immunogenicity

Six-week-old female CD-1/ICR mice were immunized by the i.m. route with 10^7^ TCID_50_ MVA-ZIKV-NS1 in 0.1 mL or phosphate buffered saline (PBS) following a prime-only or a prime-boost regimen, respectively. Mice in the prime-only regimen were immunized once on Day 0, while those in the prime-boost regimen were immunized on Days 0 and 28. To assess the potential that pre-existing immunity from previous vaccination with an MVA-vectored vaccine could interfere with immunogenicity from subsequent immunization, groups of eight 6-week-old CD-1/ICR mice were immunized with 1 × 10^7^ TCID_50_ MVA-ZIKV-NS1 or PBS. All mice were bled 4 weeks later and were vaccinated with 1 × 10^7^ TCID_50_ MVA-EBOV (Fig. 5d). Mice were terminally bled by cardiac puncture 4 weeks later for serum isolation. All protocols and practices for the handling and manipulation of animals were approved by the IACUC at the Centers for Disease Control and Prevention and were in accordance with the guidelines of the American Veterinary Medical Association (AVMA) for humane treatment of laboratory animals.

### ELISA

Whole blood samples were obtained by cheek puncture just prior to immunization, at Days 14 and 28 after each vaccination, and on Day 24 after challenge. Sera were obtained by centrifugation of the whole blood samples in serum separator tubes at 3500-xg for 5 min. Nunc-Maxisorp flat-bottom 96-well plates (ThermoFisher Scientific) were coated overnight at 4° C with either ZIKV NS1 protein (Sino Biological) at 1 μg/mL in PBS, recombinant Ebola virus glycoprotein (IBT Bioservices) at 1 μg/mL in PBS, or ZIKV Cos-1 prME antigen at 1:800 in bicarbonate buffer. Plates were washed 4 times with phosphate buffered saline (PBS) +0.05% Tween-20 (PBST). Next, the plates were blocked with StartingBlock Block Buffer (ThermoFisher Scientific) for 5 minutes at room temperature. Serially diluted heat-inactivated mouse serum samples were added to wells and incubated for 1 hour at 37° C. After washing with PBST, goat anti-mouse IgG-HRP (ImmunoReagents) was added to each well and incubated for 1 hour at 37° C. After a final washing, SureBlue TMB (3,3′, 5, 5′ – Tetramethylbenzidine) 1-component substrate solution (KPL) was added to each well, incubated in the dark at room temperature for 10 minutes, and stopped with 1 N hydrochloric acid (HCl). Optical density at 450 nm was determined using a Vmax Kinetic ELISA Absorbance Microplate Reader (Molecular Devices). Normalized absorbance values were determined by first subtracting optical density values of blank negative control wells. Endpoint titer was determined by the dilution at which the optical density equaled 0.03 (Fig. 1c,d).

### Serum isotyping assay

To investigate the antibody isotype that predominates the anti-NS1 humoral response, an NS1-antigen specific isotyping assay was performed by adapting a commercially available mouse monoclonal antibody isotyping kit (Sigma-Aldrich). ELISA plates were coated overnight at 4° C with cell lysate of ZIKV-infected Vero cells. The plates were washed with PBST, then blocked for 1 hour at room temperature with PBS + 1% BSA (PBSB) buffer. Immune sera were diluted 1:1000 in PBSB buffer, applied to the plate, and incubated 1 hour at 37° C. Plates were washed as previously, then incubated with 1:1000 dilution in PBSB of goat anti-mouse-IgG1, -IgG2a, -IgG2b, -IgG3, -IgM, or -IgA, and incubated for 1 hour at room temperature. Following washing, the plates were incubated with 1:5000 dilution in PBSB of horseradish peroxidase (HRP)-conjugated rabbit anti-goat Ig (Sigma-Aldrich), and incubated at room temperature for 30 minutes. After washing, SureBlue TMB substrate solution (KPL) was applied to the wells of the plate and incubated 15 minutes at room temperature, quenched with an equal volume 1 N HCl, and OD (405 nm) was measured on a Vmax plate reader (Molecular Devices) (Fig. 4a).

### Protective Efficacy

A representative African (MR766) ZIKV strain was employed as a challenge strain based on previously demonstrated age-independent mortality of CD-1/ICR mice exposed i.c.^19^. The MR766 virus is the prototype isolate from Uganda from 1947, and had been passaged up to 149 times in suckling mouse brain and twice in Vero cell culture. Groups of eleven previously singly age-matched immunized or naïve (PBS-immunized control) female CD-1/ICR mice (Charles River) were inoculated i.c. following isoflurane anesthesia in the right brain hemisphere with a 30-gauge needle affixed to a Hamilton syringe sheathed by a pipette tip allowing no more than a 4-mm needle penetrance into the skull cavity. For the i.c. inoculations, 5 log_10_ plaque forming units (PFU) diluted in PBS or PBS alone were administered in a 10 µL inoculum. Inoculated mice were placed back in their cages and monitored for recovery from anesthesia. All inoculated mice were monitored twice daily for clinical signs of morbidity (e.g. incoordination, ataxia, limb weakness/paralysis or weight loss ≥15% body weight) (Fig. 2a,b). Upon observation of morbidity in any group, monitoring was increased to four times daily. Any mouse that was identified with clinical signs that precluded ambulation or demonstrated >15% weight loss criteria was euthanized by isoflurane anesthesia followed by cervical dislocation (Fig. 5a–c). Just prior to euthanasia, mice were bled by cardiac puncture. Following cervical dislocation, brains were removed and weighed. Viral titers from the brains were determined by homogenizing brain tissue in BA-1 media using a pestle, clarifying by centrifugation, and plaque titrating on Vero cells as described previously^25^. All animal studies were conducted under approved IACUC protocols at the Centers for Disease Control and Prevention. In a separate experiment, three mice from both the PBS and the single MVA-ZIKV-NS1 immunization groups were sacrificed as described above at 5 days post infection and brain titers were determined as described above (Fig. 5c). Additionally, 15 female CD-1/ICR mice that were given two vaccinations at 0 and 28 days and 8 CD-1/ICR PBS immunized mice were challenged by i.c. inoculation and followed as described above (Fig. 2b,d).

### Plaque Reduction Neutralization assay

A plaque double-overlay assay was performed to determine neutralizing titers of mouse serum. Briefly, sera were heat-inactivated at 56° C for 30 minutes, serially diluted two-fold in 96 well plates, and 100 PFU of ZIKV strain PRVABC59 was added to an equal volume of each serum dilution. After incubating at 37° C for 2 hours, the serum/virus mixture was used to inoculate confluent Vero cells in 6 well plates. After 1 hour, an agar overlay was added to each well and cells were incubated at 37° C. On Day 3 post-inoculation, a second agar overlay containing neutral red was added to each well. Plaques were counted on Day 4 post-inoculation. PRNT_50_ was determined as the reciprocal of the serum dilution that inhibited ≥50% of the tested ZIKV inoculum (Fig. 3b).

### ADCC assay

Sera from vaccinated or control mice were tested for ADCC activity against ZIKV-infected Vero cells using an ADCC Reporter Bioassay (Promega), following the manufacturer’s guidelines. In summary, Vero cells were grown to 90–95% confluency and inoculated with ZIKV (strain PRVABC59) at an MOI of 0.1 in DMEM medium +2% FBS. The cells were incubated at 37° C with 5% CO_2_ for 48 hours, then trypsinized, washed with PBS, and resuspended in ADCC assay buffer. A small aliquot of the cells was set aside to assay for the presence of cell surface NS1 antigens by flow cytometric analysis using MVA-ZIKV-NS1 sera (pooled from Day 28 post-boost mouse sera) and mouse monoclonal anti-ZIKV NS1 protein (Aalto Bio Reagents) as a control (Fig. 4b). Mouse sera were serial diluted in ADCC assay buffer with a starting dilution of 1:10. 1.25 × 10^4^ ZIKV-infected Vero target cells were plated into individual wells of a 96-well plate together with 7.5 × 10^4^ effector cells, for an effector-to-target ratio of 6:1. The effector cells consisted of a Jurkat cell line stably expressing FcγRIIIa and expressing luciferase behind an NFAT promoter. In this bioassay ADCC activity is read out as a function of luciferase activity in the effector cells. After plating the cells, the serially-diluted sera were added to the wells, gently mixed, and the plate was placed at 37° C with 5% CO_2_ for 6 hours. The plate was then equilibrated to room temperature for 15 minutes. Bio-Glo luciferase Assay Reagent (Promega) was added to the wells, incubated for 10 min at room temperature, and finally luminescence was measured using a SpectraMax L plate reader (Molecular Devices) set with an acquisition time of 0.1 seconds. Sera from mice immunized with adenovirus expressing ZIKV prME were used as a control. Endpoint titers were calculated as the reciprocal of the lowest serum dilution that yielded a signal above the mean +2 standard deviations of naïve sera samples (Fig. 4c).

### Complement-mediated cellular cytotoxicity assay

Vero cells infected with ZIKV (strain PRVABC59) for 48 hours with an MOI of 0.1 were used as target cells to measure complement-mediated cellular cytotoxicity. The cells were incubated at 37° C in the presence of 1:20 dilution of pooled anti-MVA-ZIKV-NS1 sera or pooled naïve sera and 1:10 dilution of rabbit complement (Pel-Freez Biologicals), or heat-inactivated rabbit complement (inactivated by treatment at 56° C for 30 minutes) and 1 µg/mL propidium iodide. After 30 minutes incubation, the cells were assayed for viability by measuring inclusion of propidium iodide by flow cytometry (BD FACSCanto). Measurements were repeated at 90 minutes, a time point chosen because previous experiments demonstrated complete specific lysis by this time. Percent specific lysis was calculated as the number of dead cells measured at 30 minutes divided by the maximal number of cells lysed at 90 minutes (Fig. 4d).

### T cell assay

CD-1/ICR mice immunized once with 1 × 10^7^ TCID_50_ MVA-ZIKV-NS1 vaccine were sacrificed 10 days after immunization, and spleens were harvested. Naïve mouse spleens were harvested in parallel. Isolated splenocytes were analyzed by intracellular cytokine staining following standard protocols. In short, 10^6^ splenocytes were stimulated with 0.1 µg ZIKV NS1 peptide library (JPT Peptide Technology), 0.1 µg HIV Env peptide library (21st Century Biochemicals) or mock stimulated. Incubation proceeded for 6 hours at 37° C +5% CO_2_ and GolgiPlug (BD Biosciences) was added at a concentration of 1.0 µL/mL for the last 4 hours of the incubation. The splenocytes were stained with Live/Dead Fixable Green Dead Cell Stain (ThermoFisher) at room temperature for 20 minutes. The samples were treated with Cytofix/Cytoperm (BD Biosciences) at 4° C for 20 minutes and were stained with CD3 APC-CY7, CD4 PE-Cy7, CD8 PerCP, IL2 PE, and IFN-γ Alexa647 (all flow cytometry antibodies from BD Biosciences). Samples were analyzed with a FACSCanto flow cytometer (BD Biosciences) utilizing FACSDiva software. Analysis was performed with FlowJo software. Cytokine responses were scored as a percent of total CD4^+^ (Fig. 4e) or CD8^+^ (Fig. 4f) T cells.

### Statistical Analyses

Analysis of data was performed using GraphPad Prism (GraphPad Software). Comparisons between one group and another were performed using a student t-test. To test for pairwise differences between groups, an analysis of variance (ANOVA) and Tukey’s test of multiple comparisons was performed. Correlations were performed by Spearman rank-correlation tests. A p value less than 0.05 was considered statistically significant.

A sample size of 8 mice was used for controls and based on previous assessment would expect to result in >2 survivors (75% virulence). We previously used 8 mice for each group, and the number was determined by using the PASS program with a two proportions power analysis. We found that a group sample size of 8 will achieve 92% power to detect a difference of 0.79 (set P1-control group at 0.01 proportion, which means that less than 1% survive; and P2-vaccinated group at 0.8 to 0.99 proportion which means we only indicate that the vaccine is efficacious if it is protective for at least 80% of vaccinated mice) between the null hypothesis that both group proportions are 0.01 and the alternative hypothesis that the proportion in group 2 is 0.8 using a 2-side Chi-square test with continuity correction and with a significance level of 0.05 (α).

Mice were randomly assigned to the different experimental groups, and no mice were excluded from the analysis. No blinding was performed. Empirical assessments of weight and clinical symptomology were utilized as endpoints as described.

### Approval

All experimental protocols were approved by the IACUC at the National Centers for Diseases Control and Prevention, Ft. Collins, Colorado.

### Accordance

All methods were carried out in accordance with the relevant guidelines and regulations.

### Data availability

All data generated or analysed during this study are included in this published article (and its Supplementary Information files).

## Electronic supplementary material

Supplementary information
