# Supplementary material for: A Zika Vaccine Targeting NS1 Protein Protects Immunocompetent Adult Mice in a Lethal Challenge Model
Source: Sci Rep. 2017 Nov 7;7:14769. doi: 10.1038/s41598-017-15039-8 (PMC5677088; doi:10.1038/s41598-017-15039-8)
Supplement: Supplementary file 1 — Supplementary information [file 41598_2017_15039_MOESM1_ESM.docx]

**SUPPLEMENTARY MATERIALS**

**Title: A Zika Vaccine Targeting NS1 Protein Protects Immunocompetent Adult Mice in a Lethal Challenge Model**

**Authors:** Aaron C. Brault^1^, Arban Domi^2^, Erin M. McDonald^1^, Dalit Talmi-Frank^1^, Nathanael McCurley^2^, Rahul Basu^2^, Harriet L. Robinson^2^, Michael Hellerstein^2^, Nisha K. Duggal^1^, Richard A. Bowen^3^, and Farshad Guirakhoo^2*^

**Affiliations:**

^1^Division of Vector-Borne Diseases, Centers for Disease Control and Prevention, Fort Collins, CO, USA.

^2^GeoVax, Inc., Smyrna, GA, USA.

^3^Department of Biomedical Sciences, Colorado State University, Fort Collins, CO, USA.

*Correspondence to: fguirakhoo@geovax.com

**SUPPLEMENTARY TEXT**

**Utility of NS1 immunization**. The idea of a flavivirus vaccine based on the NS1 protein is not new; but there has not been an incentive, at least until now, to further develop such a vaccine beyond the preclinical stages, since the standard approaches (e.g. PIV or LAV) using prME immunogens have been quite adequate in generating effective vaccines against TBE, JEV, yellow fever and DENV(*1*). Only recently, due to the ADE concern (ZIKV and DENV have the highest sequence identity of all flaviviruses(*2*), efforts are being directed toward alternative vaccines (e.g. using a stabilized E protein without the ADE epitopes, E protein-derived domain III, or an NS1 approach) against both ZIKV and DENV (personal communications). For development of an alternative NS1 based-DENV vaccine, however, it may be necessary to delete or mutate the NS1 a.a. residues 305-311 (GKLITEW), which has 75% identity with human plasminogen (residue 590-597) (GTLISPEW), to avoid potential induction of plasminogen cross-reactive antibodies that has been suggested, through molecular mimicry, to contribute to pathogenesis of dengue hemorrhagic fever and dengue shock syndrome(*3*).  Vaccination of inbred C3H/HeN mice with a modified DENV2 NS1 protein (deletion of the C-terminal a.a. 271-352 of DEN2 or replacement with the corresponding JEV NS1 a.a, reduced its pathology(*4*).  Nevertheless, immunization with a native NS1, fully protected IFN receptor-deficient mice from lethal challenge with DENV-2 without induction of any vascular leakage(*5*).  In contrast to cross reactivity of E specific antibodies among flaviviruses, especially between DENV serotypes and now between DENV and ZIKV, a concern for ADE(*6, 7*), no cross reactivity was found among antibodies directed to NS1 proteins in humans infected with ZIKA and DENVs(*8*). Moreover, the molecular mimicry between NS1 epitopes and human plasminogen, seems to be limited to DENVs and no NS1-induced pathology has been reported for any other flaviviruses so far, eliminating any safety concerns over using native NS1 as an effective alternative immunogen for ZIKV immunization.

**SUPPLEMENTARY REFERENCES**

1. T. Ishikawa, A. Yamanaka, E. Konishi, A review of successful flavivirus vaccines and the problems with those flaviviruses for which vaccines are not yet available. *Vaccine*, (2014).

2. M. Xu *et al.*, Identification of small-molecule inhibitors of Zika virus infection and induced neural cell death via a drug repurposing screen. *Nat Med*, (2016).

3. Y. C. Chuang, J. Lin, Y. S. Lin, S. Wang, T. M. Yeh, Dengue Virus Nonstructural Protein 1-Induced Antibodies Cross-React with Human Plasminogen and Enhance Its Activation. *J Immunol* **196**, 1218-1226 (2016).

4. S. W. Wan *et al.*, Protection against dengue virus infection in mice by administration of antibodies against modified nonstructural protein 1. *PLoS One* **9**, e92495 (2014).

5. P. R. Beatty *et al.*, Dengue virus NS1 triggers endothelial permeability and vascular leak that is prevented by NS1 vaccination. *Science translational medicine* **7**, 304ra141 (2015).

6. F. X. Heinz, K. Stiasny, The Antigenic Structure of Zika Virus and Its Relation to Other Flaviviruses: Implications for Infection and Immunoprophylaxis. *Microbiol Mol Biol Rev* **81**, (2017).

7. L. M. Paul *et al.*, Dengue Virus Antibodies Enhance Zika Virus Infection. *bioRxiv* **posted online**, (2016).

8. K. Stettler *et al.*, Specificity, cross-reactivity, and function of antibodies elicited by Zika virus infection. *Science* **353**, 823-826 (2016).
